# Effects of CAR-T Cell Therapy on Immune Cells and Related Toxic Side Effect Analysis in Patients with Refractory Acute Lymphoblastic Leukemia

**DOI:** 10.1155/2023/2702882

**Published:** 2023-06-03

**Authors:** Lianlian Li, Jie Gao, Zhaojun Sun, Xiaolei Li, Ning Wang, Rui Zhang

**Affiliations:** Department of Hematology, Cangzhou People's Hospital, Cangzhou City, Hebei Province, China

## Abstract

**Objective:**

To observe the effects of chimeric antigen receptor T (CAR-T) cell immunotherapy on immune cells and related toxic side effects in patients with refractory acute lymphoblastic leukemia (ALL).

**Methods:**

A retrospective study was conducted in 35 patients with refractory ALL. The patients were treated with CAR-T cell therapy in our hospital from January 2020 to January 2021. The efficacy was evaluated at one and three months post treatments. The venous blood of the patients was collected before treatment, 1 month after treatment, and 3 months after treatment. The percentage of regulatory T cells (Treg cells), natural killer (NK) cells, and T lymphocyte subsets (CD3+, CD4+, and CD8+ T cells) was detected by flow cytometry. The ratio of CD4+/CD8+ was calculated. Patient's toxic side effects such as fever, chills, gastrointestinal bleeding, nervous system symptoms, digestive system symptoms, abnormal liver function, and blood coagulation dysfunction were monitored and recorded. The incidence of toxic and side effects was calculated, and the incidence of infection was recorded.

**Results:**

After one month of CAR-T cell therapy in 35 patients with ALL, the efficacy evaluation showed that complete response (CR) patients accounted for 68.57%, CR with incomplete hematological recovery (CRi) patients accounted for 22.86%, and partial disease (PD) patients accounted for 8.57%, and the total effective rate was 91.43%. In addition, compared with that before treatment, the Treg cell level in CR+CRi patients treated for 1 month and 3 months decreased prominently, and the NK cell level increased dramatically (*P* < 0.05). Compared with that before treatment, the levels of CD3+, CD4+, and CD4+/CD8+ in patients with CR+CRi in the 1-month and 3-month groups were markedly higher, and the levels of CD4+/CD8+ in the 3-month group were memorably higher than those in the 1-month group (*P* < 0.05). During CAR-T cell therapy in 35 patients with ALL, fever accounted for 62.86%, chills for 20.00%, gastrointestinal bleeding for 8.57%, nervous system symptoms for 14.29%, digestive system symptoms for 28.57%, abnormal liver function for 11.43%, and coagulation dysfunction for 8.57%. These side effects were all relieved after symptomatic treatment. During the course of CAR-T therapy in 35 patients with ALL, 2 patients had biliary tract infection and 13 patients had lung infection. No correlations were found between the infection and age, gender, CRS grade, usage of glucocorticoids or tocilizumab, and laboratory indicators such as WBC, ANC, PLT, and Hb (*P* > 0.05).

**Conclusion:**

CAR-T cell therapy had a good effect on patients with refractory ALL by regulating the immune function of the body via mediating the content of immune cells. CAR-T cell therapy may have therapeutic effect on refractory ALL patients with mild side effects and high safety.

## 1. Introduction

Acute lymphoblastic leukemia (ALL) is a clinically common hematological tumor, accounting for about 20% to 30% of acute leukemia in adults. Clinical manifestations of ALL include the inhibition of bone marrow hematopoietic function and the proliferation and infiltration of leukemia cells, etc. ALL has a high recurrence rate and poor prognosis, which seriously affects the life of patients [1, 2]. The incidence rate of ALL is high, accounting for 15% of leukemia and about 35% of acute leukemia. At present, the main clinical treatment of ALL is chemotherapy and/or hematopoietic stem cell inhibition therapy. However, the recurrence rate and mortality rate of patients are still at a high level. Previous studies have concluded that chemotherapy has a two-year disease-free survival rate of 39.0% and a two-year overall survival rate of 58.4% for patients with ALL [3, 4]. How to improve the prognosis of refractory ALL patients has become the focus of current medical research.

Chimeric antigen receptor T (CAR-T) cell is a T cell with the ability to recognize and kill tumors, and key cytokines such as IL-12 could be expressed on the basis of whose organizational structure. Intensifying the activation reaction of T cells has a good effect in the treatment of ALL and can improve the long-term survival rate of patients by enhancing the immune response [5, 6]. In recent years, foreign scholars believe that CAR-T cell therapy has shown good efficacy in children with refractory ALL, with tolerable safety, high response rate, and excellent persistence [7]. However, the effects of CAR-T cells on immune cells in patients with refractory ALL have been reported less.

In this study, 35 patients with refractory ALL admitted in our hospital during January 2020 to January 2021 were chosen as subjects, aiming to analyze the effects of CAR-T immunotherapy on immune cells and related toxic side effects in patients with refractory ALL.

## 2. Materials and Methods

### 2.1. General Materials

**Figure 1:**
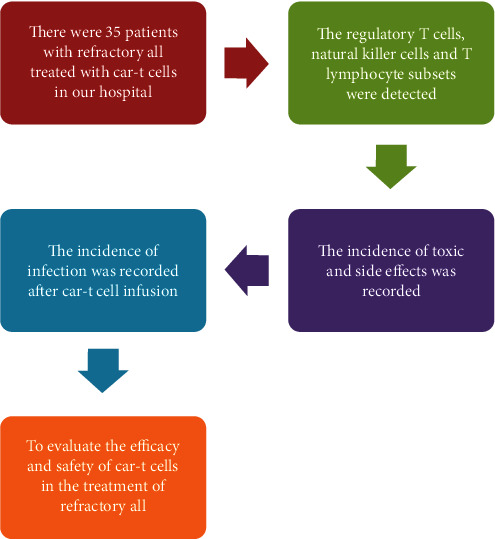
The experimental process.

Thirty-five patients with refractory ALL during January 2020 to January 2021 were chosen as subjects. The clinical data of the patients were collected and retrospectively analyzed. Inclusion criteria were as follows: (1) all patients were initially diagnosed patients who failed to respond to two standard protocols or ALL patients who recurred within 12 months after consolidation and intensive treatment after CR or who recurred twice or more [8]. (2) The patient's age was between 18 and 65 years old. (3) The patients and their family members were informed and had good compliance and could cooperate with the examination and treatment. All of them signed an informed consent form. Exclusion criteria were as follows: (1) patients with severe cardiovascular and cerebrovascular diseases, (2) patients with nervous system diseases, (3) patients with other malignant tumors, and (4) patient complicated with infection. The subjects included 18 males and 17 females, with an average age of 38.16 ± 6.85 years. The operation of this experiment was approved by the hospital Ethics Association. The experimental process is shown in Figure 1.

### 2.2. Methods

To prepare CAR-T cells, 40-60 mL of peripheral blood was collected from experimental subjects, anticoagulation with heparin: T lymphocytes were activated and expanded after isolation and purification, and CAR-T cells were amplified again after specific CAR transfection. CAR-T cells were frozen after quality inspection. CAR-T cells were infused intravenously for the treatment. All patients received chemotherapy about 30 days before CAR-T cell infusion. Appropriate chemotherapy programs were chosen by physicians according to the patient's condition and previous treatment to reduce the tumor load of patients, aiming to prevent the occurrence of cytokine release syndrome (CRS) or reduce the severity of CRS. Detect the recovery of patient's blood routine. About 3 days before cell infusion, fludarabine (Flu)+cyclophosphamide (CY) pretreatment scheme (FC) was given as follows: Flu 25 mg/m^2^ and CY 300 mg/m^2^/d, continuously injected intravenously for 3 days. According to the existing literature report in the United States [7], in the CAR-T cell reinfusion therapy, the amount of CAR-T cell reinfusion should be 10^6^-10^7^ cells/kg body weight. According to the number of T cells collected from the patient, the reinfusion plan was formulated as appropriate, and the infusion volume was 10^6^-10^7^ cells/kg body weight.

### 2.3. Outcome Measures

#### 2.3.1. Efficacy Analysis

The efficacy evaluation included complete response (CR), CR with incomplete clinical recovery (CRi), and partial disease (PD). Among them, CR represented the recovery of bone marrow hematopoiesis, with primitive cells < 5%, no primitive cells in peripheral blood, absolute value of platelets > 100 × 10^9^/L, absolute value of neutrophil > 1 × 10^9^/L, and no recurrence occurs in 4 weeks. CRi referred to the recovery of bone marrow hematopoiesis, with primitive cells < 5% and no primitive cells in peripheral blood, but the absolute value of patient's platelet ≤ 100 × 10^9^/L, absolute value of neutrophil ≤ 1 × 10^9^/L, and no recurrence in 4 weeks. PD represented 25% increase of primitive cells found in bone marrow or peripheral blood, or extramedullary infiltration occurs.

#### 2.3.2. Detection of Serum Indicators

The venous blood of patients was collected before treatment, 1 month after treatment, and 3 months after treatment and stored in heparin anticoagulant tubes. After centrifugated at 1500 r/min for 5 min, the supernatant was carefully separated and stored into the refrigerator at -80°C to avoid repeated freezing and thawing. The percentage of regulatory T (Treg) cells, natural killer (NK) cells, and T lymphocyte subsets CD3+, CD4+, and CD8+ T cells was detected by TUNEL Flow Cytometry Analysis Kit (purchased from Wuhan Purity Biotechnology Co., Ltd., Hongshan District, Wuhan, Hubei Province, China) and HLA-B27 Assay Kit (purchased from Guangzhou Jincheng Biotechnology Co., Ltd., Tianhe District, Guangzhou, Guangdong Province, China). The ratio of CD4+/CD8+ T cells was calculated.

#### 2.3.3. Toxic and Side Effects

Closely observe the patient's condition changes and monitor and record the patient's toxic and side effects such as fever, chills, gastrointestinal bleeding, nervous system symptoms (dizziness, headache, irritability, aphasia, photophobia, etc.), digestive system symptoms (vomiting, nausea, etc.), abnormal liver function, and blood coagulation dysfunction. The incidence of side effects was calculated.

#### 2.3.4. Infection

After CAR-T cell infusion treatment, the presence of infection was comprehensively judged according to laboratory indicators, imaging, histopathology, and/or microbiological examination. Infection within 30 days after infusion requires intravenous antibiotics or hospitalization when severe infection occurs. The age, gender, cytokine release syndrome (CRS) grade (Table 1), whether there was a usage of glucocorticoid and tocilizumab, and laboratory indicators including white blood cell (WBC), neutrophil (ANC), platelet count (PLT), and hemoglobin (Hb) were collected.

### 2.4. Statistical Analysis

SPSS 20.0 software was used to analyze the experimental data. Measurement data such as Treg cells, NK cells, CD3+, CD4+, and CD4+/CD8+ were represented as ($\bar{x}\pm s$). Repeated measurement ANOVA was used for comparison among groups, and these with statistical differences were further compared with the Tukey test. The SNK-q test was used for comparison of multiple sample averages between groups. Enumeration data such as curative effect and adverse reaction were expressed as %, and *χ*^2^ test was used for comparison between groups. *P* < 0.05 indicated that the statistical results were statistically significant.

## 3. Results

### 3.1. Analysis of Curative Effect after Treatment

**Figure 2:**
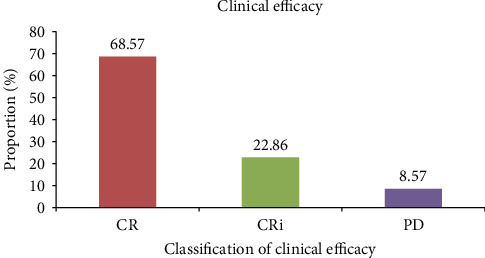
Analysis of curative effect after treatment.

After one month of CAR-T cell therapy in 35 patients with ALL, the efficacy evaluation showed that CR patients accounted for 68.57% (24 cases), CRi patients accounted for 22.86% (8 cases), and PD patients accounted for 8.57% (3 cases), and the total effective rate was 91.43% (Figure 2).

### 3.2. Comparison of Treg Cell Level before and after Treatment

The patients were grouped according to the time after treatment, and the Treg cell level was detected. Compared with that before treatment, the Treg cell level in CR+CRi patients treated for 1 month and 3 months decreased prominently, and the NK cell level increased dramatically (*P* < 0.05, Table 2).

### 3.3. Comparison of T Lymphocyte Subsets before and after Treatment

Compared with that before treatment, the levels of CD3+, CD4+, and CD4+/CD8+ in patients with CR+CRi in the 1-month and 3-month groups were markedly higher, and the levels of CD4+/CD8+ in the 3-month group were memorably higher than those in the 1-month group (*P* < 0.05, Table 3).

### 3.4. Analysis of Side Effects after Treatment

**Figure 3:**
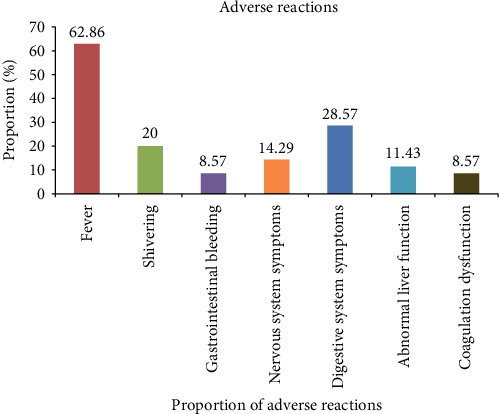
Analysis of side effects after treatment.

During CAR-T cell therapy in 35 patients with ALL, fever accounted for 62.86%, chills for 20.00%, gastrointestinal bleeding for 8.57%, nervous system symptoms for 14.29%, digestive system symptoms for 28.57%, abnormal liver function for 11.43%, and coagulation dysfunction for 8.57%. These side effects were all relieved after symptomatic treatment (Figure 3).

### 3.5. Infection Analysis

During the course of CAR-T therapy in 35 patients with ALL, 2 patients had biliary tract infection and 13 patients had lung infection. No correlations were found between the infection and age, gender, CRS grade, usage of glucocorticoids or tocilizumab, and laboratory indicators such as WBC, ANC, PLT, and Hb (*P* > 0.05, Table 4).

## 4. Discussion

ALL is a common malignant hematological disease, accounting for 80% of acute leukemia in children. At present, the clinical treatment effect of ALL is good with the CR rate of as high as 70%-90%, but some patients show refractory ALL or easy to relapse. About 30% of ALL patients have relapses after conventional induction remission, and the cure rate of refractory or relapses is low, only about 5% [9, 10]. Therefore, how to improve the cure rate and quality of life of patients with refractory ALL has become the focus of current research.

CAR-T cell therapy is to transform T cells into T cells carrying a single-chain variable fragment (scFV) with specific extracellular recognition antigen by genetic engineering, which has the function of targeting to kill tumor cells. After *in vitro* expansion, CAR-T cells are transfused back to patients to improve their immune function and kill abnormal leukemia cells in the body, which can help improve the life quality and prolong the lives of patients with ALL [11, 12]. As a new immunotherapy, CAR-T cell therapy provides a treatment option for hematologic malignancies. At the same time, some scholars are studying the application value of CAR-T cell therapy in solid tumors. Berdeja et al. [13] have concluded that the CAR-T cell assay shows a high-quality response, and a single cilta-cell infusion at a target dose of 0.75 × 10^6^ CAR-positive live T cells per kilogram can produce an early, deep, and durable response in patients with multiple myeloma who have undergone extensive pretreatment, with a controllable safety profile. In this study, after one month of CAR-T cell therapy, the efficacy evaluation showed that CR patients accounted for 68.57%, CRi patients accounted for 22.86%, and PD patients accounted for 8.57%, with the total effective rate of 91.43%. During the CAR-T cell therapy, fever accounted for 62.86%, chills for 20.00%, gastrointestinal bleeding for 8.57%, nervous system symptoms for 14.29%, digestive system symptoms for 28.57%, abnormal liver function for 11.43%, and coagulation dysfunction for 8.57%. These side effects were all relieved after symptomatic treatment. These results suggested that CAR-T cells were effective in the treatment of refractory ALL without serious side effects. After symptomatic treatment, the side effects were relieved with high safety, which was similar to the research results of Li and Chen [14].

Studies have proved that the occurrence and development of ALL are closely related to the changes of immune function [15, 16]. The change of cellular immunity is more closely related to ALL. Among them, Treg cells, NK cells, and T lymphocyte subsets can mediate the change of cellular immune function. Treg cells can regulate the body's peripheral tolerance monitoring and autoimmune response, so leukemia cells will be regarded as normal cells, and the effect of immunotherapy will be weakened by inhibiting specific antitumor T cells [17, 18]. Niu et al. [19] believed that the increase of Treg cell level indicated the failure of ALL treatment or recurrence. NK cells have cytotoxic function and immune regulation function and are the first line of defense against infection and tumor. After a period of effective treatment for ALL, the NK cell level gradually recovers and the immune function of the body is enhanced [20]. T lymphocyte subsets mainly include CD3+, CD4+, and CD8+. Among them, the level of CD3+ reflects the number of T lymphocytes in the body, CD4+ determines the change of immune cell function in the body, and CD8+ is an immunosuppressive factor. Therefore, the ratio of CD4+/CD8+ reflects the changes of cellular immune function [21]. In this study, compared with that before treatment, the Treg cell level in CR+CRi patients treated for 1 month and 3 months decreased prominently, and the NK cell level increased dramatically. Compared with that before treatment, the levels of CD3+, CD4+, and CD4+/CD8+ in patients with CR+CRi in the 1-month and 3-month groups were markedly higher, and the levels of CD4+/CD8+ in the 3-month group were memorably higher than those in the 1-month group. It was suggested that CAR-T cell therapy improved the cellular immune function of the body by mediating the changes of Treg cells, NK cells, and T lymphocyte subsets, which was helpful to control the patient's condition. During the course of CAR-T therapy in 35 patients with ALL, 2 patients had biliary tract infection and 13 patients had lung infection. In addition, no correlations were found between the infection and the age, gender, CRS grade, usage of glucocorticoids or tocilizumab, and laboratory indicators such as WBC, ANC, PLT, and Hb. No related factors of infection were found in the present study. However, some reports indicated that [22, 23] the more severe CRS after CAR-T cell treatment was, the greater the possibility of infection was. After infusion of CAR-T cells, it is sometimes difficult to distinguish infection and CRS reaction. Thus, the prevention, diagnosis, and treatment of infection after CAR-T treatment need further research. Studies have also found that early stage after CAR-T infusion is affected by pretreatment by depigmentation, and later stage is related to cytokine-mediated cytopenias. Some patients may experience prolonged cytopenia and require blood transfusion or growth factor support, during which the patient's immune function is significantly reduced and the infection rate increases [24, 25]. The absence of infectious factors that was identified in this study may be due to the small number of cases included in this study and the single-centre retrospective study, which might have data bias.

In general, CAR-T cell therapy had a good effect on patients with refractory ALL by regulating the immune function of the body via mediating the content of immune cells. This therapy may have a therapeutic effect on refractory ALL patients with mild side effects and high safety. However, due to the limitation of research time and sample size in this experiment, the long-term prognosis of patients was not followed up and there was no control group. In the future, the mechanism of Treg cells influencing the curative effect of CAR-T cells will be further clarified through *in vivo* and *in vitro* experiments. We need to verify whether the therapeutic effect of CAR-T cells can be improved and the recurrence can be reduced by intervening Treg cells. Therefore, the sample size and research time will be expanded for in-depth exploration in our following study.

## Figures and Tables

**Table 1:** CRS grade.

Indicators	Grade 1	Grade 2	Grade 3	Grade 4
Fever	Body temperature ≥ 38°C	Body temperature ≥ 38°C	Body temperature ≥ 38°C	Body temperature ≥ 38°C
Hypotension	No	No need to use antihypertensive drugs	1 antihypertensive drug is required, with or without vasopressin	Multiple antihypertensive drugs are required (excluding vasopressin)
		And/or		
Hypoxemia	No	Need low flow nasal catheter oxygen inhalation	Need high flow nasal catheter oxygen inhalation and circulatory respirator	Need positive pressure ventilation

**Table 2:** Comparison of Treg cell level before and after treatment ($\bar{x}\pm s$).

Groups	Treg cells (%)	NK cells (%)
Before treatment (*n* = 35)	8.16 ± 2.86	11.78 ± 0.62
CR+CRi patients treated for 1 month (*n* = 32)	7.05 ± 1.25^a^	13.25 ± 0.56^a^
CR+CRi patients treated for 3 months (*n* = 32)	6.15 ± 2.13^ab^	15.02 ± 0.32^ab^
PD patients treated for 1 month (*n* = 3)	10.85 ± 1.16^bc^	11.64 ± 0.89
*F*	7.500	17.770
*P*	<0.001	<0.001

^a^ *P* < 0.05 compared with before treatment; ^b^*P* < 0.05 compared with CR+CRi patients treated for 1 month; ^c^*P* < 0.05 compared with CR+CRi patients treated for 3 months.

**Table 3:** Comparison of T lymphocyte subsets before and after treatment ($\bar{x}\pm s$).

Groups	CD3+ (%)	CD4+ (%)	CD8+ (%)	CD4+/CD8+ (%)
Before treatment (*n* = 35)	66.52 ± 9.12	31.95 ± 9.82	31.56 ± 7.45	1.01 ± 0.45
CR+CRi patients treated for 1 month (*n* = 32)	72.45 ± 8.56^a^	38.45 ± 8.45^a^	30.79 ± 5.26	1.25 ± 0.26^a^
CR+CRi patients treated for 3 months (*n* = 32)	73.26 ± 8.66^a^	42.59 ± 8.75^a^	29.62 ± 5.12	1.44 ± 0.31^ab^
PD patients treated for 1 month (*n* = 3)	67.45 ± 12.05	32.15 ± 4.56	31.05 ± 12.45	1.04 ± 0.25
*F*	4.010	8.340	0.540	8.700
*P*	0.010	<0.001	0.657	<0.001

^a^ *P* < 0.05 compared with before treatment; ^b^*P* < 0.05 compared with CR+CRi patients treated for 1 month.

**Table 4:** Analysis of infection after CAR-T treatment.

Groups	No infection occurs (*n* = 20)	Infection occurs (*n* = 15)	*t/χ* ^2^	*P*
Age (year)	35.25 ± 15.48	31.26 ± 12.33	0.821	0.418
Gender				
Male	8 (40.00)	10 (66.67)	2.440	0.118
Female	12 (60.00)	5 (33.33)		
CRS grade				
Grade 1	7 (35.00)	3 (20.00)	2.426	0.489
Grade 2	6 (30.00)	5 (33.33)		
Grade 3	6 (30.00)	4 (26.67)		
Grade 4	1 (5.00)	3 (20.00)		
Usage of glucocorticoids				
Yes	11 (55.00)	9 (60.00)	0.088	0.767
No	9 (45.00)	6 (40.00)		
Usage of tocilizumab				
Yes	15 (75.00)	11 (73.33)	0.013	0.911
No	5 (25.00)	4 (26.67)		
Initial WBC (×10^9^/L)	4.52 ± 1.85	4.43 ± 1.97	0.139	0.891
Initial ANC (×10^9^/L)	2.53 ± 0.28	3.05 ± 1.26	1.796	0.082
Initial PLT (×10^9^/L)	127.49 ± 10.23	131.25 ± 15.48	0.865	0.393
Initial Hb (×10^9^/L)	102.25 ± 20.42	97.26 ± 18.48	0.745	0.462

## Data Availability

The datasets used and/or analyzed during the current study are available from the corresponding author on reasonable request.
